# Postimplantation Whole Embryo Culture Assay for Hamsters: An Alternative to Rat and Mouse

**DOI:** 10.1100/tsw.2001.48

**Published:** 2001-05-25

**Authors:** Bogdan Wlodarczyk, Bogumil Biernacki, Maria Minta, Jan Zmudzki

**Affiliations:** Department of Pharmacology and Toxicology, National Veterinary Research Institute, Pulawy, Poland

**Keywords:** Syrian hamster, whole-embryo culture, embryotoxicity, teratogenicity, valproic acid, cadmium chloride, diethylstilbestrol

## Abstract

Postimplantation whole embryo culture (WEC) assay for rats and mice has been well established and introduced to many laboratories. Recently WEC technique for rabbits has been developed; however, information on culture of other species is very limited. Knowing the usefulness of hamsters in classical embryotoxicology, we reasoned that hamster WEC could be an alternative model for the most frequently used rat and mouse WEC. Previously we have optimized culture conditions for postimplantation hamster embryos. The aim of this study was to test the susceptibility of hamster embryos cultured in vitro to embryotoxic compounds and to compare our results with those reported by others on rat or mouse embryo culture. For that purpose we choose three known embryotoxic compounds--valproic acid, cadmium chloride, and diethylstilbestrol--and tested them using a postimplantation hamster whole embryo culture assay. Hamster embryos were cultured from 7.5 days gestation for 24 h in a medium consisting of 35% hamster serum and 65% synthetic culture medium (Iscove's or McCoy 5A). At the end of the culture period, the embryos were examined morphologically, measured with the aid of a computer image analysis system, and total protein content was assessed. All three compounds exhibited dose-related embryotoxic and teratogenic effects in hamster embryos. The malformations observed were similar to those reported on rat and mouse embryos. Comparison of the results with data reported by other authors indicates that hamster embryos cultured in vitro might be more susceptible to embryotoxic stimuli than rat and mouse embryos.

## INTRODUCTION

Rodent whole embryo culture (WEC), developed originally by New and his associates [1] in the 1960s, is now used in many laboratories worldwide for evaluation of the mechanisms of development and teratogenesis. The whole embryo culture is also frequently applied as a pre-screen test to detect embryotoxic potential of new chemical compounds, even though the final results of validation studies of WEC founded by the European Center for the Validation of Alternative Methods are not yet published. [2]. Preliminary data from the validation studies indicate that the prediction model to identify embryotoxic chemicals for the WEC test amounts to 79% and when combined with the cytotoxicity data from embryonic stem cell test results in 96% correct classifications [3]. Most frequently rat embryos are used in postimplantation embryo culture, although a few laboratories are also using mouse embryos. Recently WEC techniques for rabbits have been developed [4,5,6].

In our laboratory, we have been using golden hamsters for teratology testing for over 30 years. The main advantages of this species include the shortest period of gestation (16 days) found among rodent laboratory animals, a fast progression through organogenesis, and a high susceptibility to teratogens *in vivo*. Given these advantages, we considered whether the hamster might provide a better alternative model to the rat or mouse whole embryo culture. To date only two attempts to culture postimplantation hamster embryos appear in the literature [7,8]. The pioneer work was done by Givelber and DiPaolo who cultured hamster embryos attached to a rayon cloth or Millipore filter in a big volume circulating medium. The survival rate of embryos after 24 h of culture was 77 to 81%. There were also big variations in size and development of the embryos as well as high incidence of malformations [7].

More recently, Ebron-McCoy and coworkers established WEC technique for hamster embryos [8]. In these studies authors tried to optimize the culture conditions using three different heterologous media: the first composed of rat serum only; the second, rat serum supplemented with synthetic culture medium; and the third, rat and fetal bovine serum supplemented with synthetic culture medium. The authors did not test homologous hamster serum because “high volume” culture (10 ml of medium per embryo) used in these studies would require sacrificing a large number of adult hamsters to obtain enough volume of serum. Additionally, the embryotoxic potentials of sodium salicylate and cyclophosphamide on hamster embryos were tested in those studies.

In order to establish hamster whole embryo culture in our laboratory, we tested the usefulness of different culture media for this purpose. Among the media tested (undiluted hamster, rat, or bovine serum; hamster and rat serum supplemented with different amounts of synthetic culture media) the best for maintaining growth of hamster conceptuses in culture was either 100% hamster serum or hamster serum supplemented with McCoy 5A or Iscove’s synthetic culture medium, where the serum concentration was at least 30% [9].

The aim of the present study was to test the susceptibility of hamster embryos cultured *in vitro* to known teratogens and to compare our results with previously reported studies that used either rat or mouse embryo culture. For this purpose, we chose well recognized and defined rodent teratogens: valproic acid, cadmium, and diethylstilbestrol [10,11,12,13,14,15,16,17,18,19,20]. Additionally, we used penicillin G, a nonterato-gen, which is frequently used as a negative control in teratological studies. We reasoned that the identification of possible differences between species in susceptibility to teratogens may be helpful in assessing the risk to humans of teratogenic agents.

## RESULTS AND DISCUSSION

### Control Embryos

At the end of the 24-h culture period (gestational day 8.5), control hamster embryos appeared morphologically normal, having completed axial rotation and exhibiting a beating heart, three branchial bars, otocysts, optic vesicles, olfactory plates, and forelimb buds. The neural tube was totally closed throughout the body axis, including both the anterior and posterior neuropores. The hindlimb buds appeared as a slight evagination at the level of somites 20–26. A full plexus of yolk sac vessels was visible and the vitelline artery and vein were often separated. The morphological score in control embryos usually was 47–50, with a somite number of 23–25 (Fig. 1).

### VPA-Treated Embryos

VPA-induced adverse effect on growth and development of hamster embryos was evident at all tested doses, especially at the higher concentrations of 300 and 600 μM, where all developmental parameters were affected. VPA-treated embryos were smaller, had fewer somites, and had lower protein content than the controls. Generally they were much less advanced in development and differentiation, a finding that was confirmed by their reduced morphological scores. The observed embryotoxic effects were dose-related and statistically significant (Table 1). The most frequently observed malformations were open neural tube in the head region (16 embryos), dilated pericardium (5), irregular somites and wavy suture line of neural folds (4), incomplete rotation (3), asymmetrically enlarged forebrain (2), and open posterior neuropore (2) (Figs. 2 and 3). Four embryos in a group treated with the highest dose of VPA were grossly retarded and remained unturned, exhibiting a “U” shape at the end of the culture period.

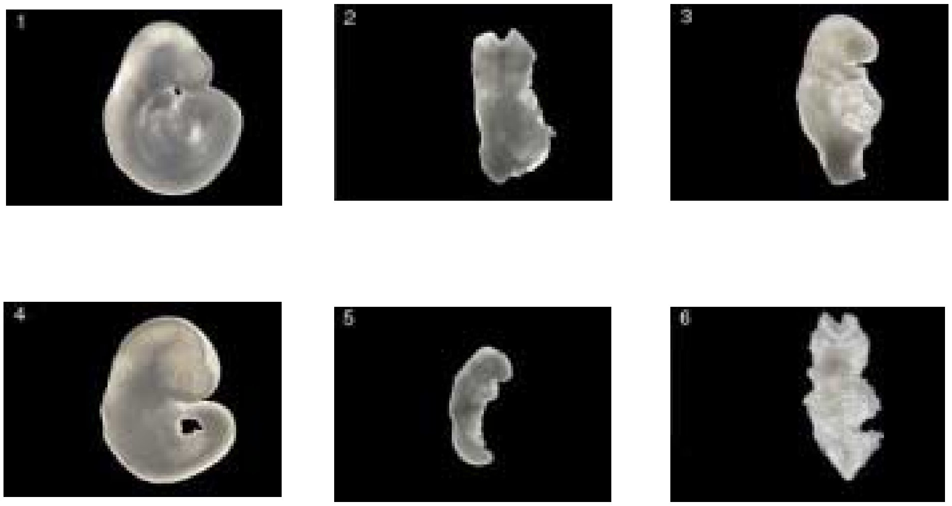

**FIGURES 1 to 6**. Hamster embryo. (1) After 24-h culture in control medium. (2) After 24-h culture in medium supplemented with VPA 600 µM. Retarded embryo, neural folds widely open along the entire body axis. (3) After 24-h culture in medium supplemented with VPA 600 µM. Slightly retarded embryo, dilated pericardium. (4) After 24-h culture in medium supplemented with cadmium chloride 0.1 µM. The cranial part of the embryo is swollen. (5) After 24-h culture in medium supplemented with DES 10 µM. Severely retarded embryo with open neural tube in mid- and hindbrain region. (6) After 24-h culture in medium supplemented with DES 10 µM. Severely retarded embryo with open neural tube in midbrain region, waviness of neural tube in spinal region, irregular and deformed somites.

**TABLE 1**
**Effects of Valproic Acid on Growth and Development of 8-day Hamster Embryos Cultured for 24 h**

| VPA concentration, µM | 0 | 60 | 300 | 600 |
|---|---|---|---|---|
| No. of embryos | 16 | 16 | 16 | 16 |
| Yolk sac diameter, mm | 4.02 ± 0.21 | 3.78 ± 0.09 | 3.51 ± 0.18* | 3.21 ± 0.24* |
| Crown-rump length, mm | 3.14 ± 0.11 | 3.11 ± 0.10 | 2.67 ± 0.13* | 2.53 ± 0.15* |
| Head length, mm | 1.41 ± 0.07 | 1.35 ± 0.04 | 1.12 ± 0.07* | 1.06 ± 0.07* |
| Somite number | 24.8 ± 0.5 | 22.5 ± 0.6* | 20.2 ± 1.1* | 21.5 ± 1.0* |
| Protein content, µg /embryo | 140 ± 9 | 145 ± 9 | 83 ± 19* | 54 ± 15* |
| Total score | 47.9 ± 1.1 | 44.6 ± 1.3 | 31.4 ± 3.8 | 27.8 ± 3.4 |
| Malformed embryos, % | 0 | 25 | 56 | 81 |

* Significantly different from control, $p < 0.05$.

## $CdCl_2$-Treated Embryos

Cadmium chloride at the highest concentration of 0.25 µM proved to be embryotoxic and teratogenic. All developmental parameters in this group were severely affected and 70% of embryos were abnormal (Table 2). The embryonic systems most frequently malformed were neural tube (open in the head region, waviness in the spinal region and hydrocephalus), incomplete axial rotation, first branchial bars (small), allantois (not fused with ectoplacental cone) and otocyst (deformed) (Fig. 4). Exposure to 0.1 µM $CdCl_2$ resulted in 64% of embryos exhibiting malformations and a statistically significant reduction in head length, protein content, and morphological score. The abnormalities in this group were very similar to those observed in the group receiving 0.25 µM cadmium. The lowest dose of $CdCl_2$ (0.02 µM) was much less toxic and affected only the protein content.

**TABLE 2**
**Effects of Cadmium Chloride on Growth and Development of 8-day Hamster Embryos Cultured for 24 h**

| $CdCl_2$ concentration, µM | 0 | 0.02 | 0.1 | 0.25 |
|---|---|---|---|---|
| No. of embryos | 14 | 14 | 14 | 17 |
| Yolk sac diameter, mm | 3.91 ± 0.07 | 3.81 ± 0.06 | 3.95 ± 0.12 | 3.28 ± 0.14* |
| Crown-rump length, mm | 3.14 ± 0.10 | 3.08 ± 0.04 | 2.98 ± 0.14 | 2.64 ± 0.08* |
| Head length, mm | 1.41 ± 0.04 | 1.34 ± 0.04 | 1.22 ± 0.09* | 1.00 ± 0.05* |
| Somite number | 23.8 ± 0.2 | 22.9 ± 0.6 | 23.4 ± 0.6 | 19.2 ± 0.8* |
| Protein content, µg /embryo | 165 ± 10 | 129 ± 7* | 123 ± 16* | 73 ± 8* |
| Total score | 47.8 ± 0.5 | 45.6 ± 1.2 | 38.1 ± 3.4 | 30.3 ± 2.6 |
| Malformed embryos, % | 0 | 7 | 64 | 70 |

* Significantly different from control, $p < 0.05$.

One embryo in this group was malformed (hydropericardium).

## DES-Treated Embryos

Exposure to 120 µM DES resulted in a 100% malformation rate: all embryos showed neural tube defects and axial rotation abnormalities, while others additionally displayed haematomas, hydropericardium, branchial bar deformities, and irregular or fused somites. Four embryos were grossly retarded and seven embryos showed multiple malformations, and their somite number was uncountable. In the group exposed to 10 µM DES, 81% of embryos showed abnormalities (Figs. 5 and 6). The abnormalities in this group included the neural tube (11 embryos), axial rotation (8), allantois (4), somites (4), and branchial bars (1). DES at 120 and 10 µM also exhibited strong embryotoxic properties: all developmental parameters were significantly decreased compared to the controls (Table 3). The lowest DES concentration (1 µM) resulted in 13% malformed embryos (two embryos with open neural tube in the forebrain region), but there was no statistically significant decrease in embryonic parameters. Hence, the embryotoxic and teratogenic effects of DES were clearly dose-related.

**TABLE 3**
**Effects of Diethylstilbestrol on Growth and Development of 8-day Hamster Embryos Cultured for 24 h**

| DES concentration, µM | 0 | 1 | 10 | 120 |
|---|---|---|---|---|
| No. of embryos | 16 | 15 | 16 | 15 |
| Yolk sac diameter, mm | 4.17 ± 0.13 | 4.06 ± 0.13 | 3.43 ± 0.07* | 3.09 ± 0.07* |
| Crown-rump length, mm | 3.26 ± 0.05 | 3.15 ± 0.11 | 2.51 ± 0.08* | 2.27 ± 0.11* |
| Head length, mm | 1.46 ± 0.03 | 1.42 ± 0.05 | 0.97 ± 0.05* | 0.80 ± 0.04* |
| Somite number | 24.5 ± 0.3 | 23.9 ± 0.5 | 19.5 ± 0.8* | 12.4 ± 2.1* |
| Protein content, µg /embryo | 167 ± 6 | 157 ± 4 | 107 ± 11* | 64 ± 5* |
| Total score | 49.1 ± 0.8 | 47.1 ± 0.7 | 30.1 ± 3.0 | 17.9 ± 2.0 |
| Malformed embryos, % | 0 | 13 | 81 | 100 |

* Significantly different from control, $p < 0.05$.

## Penicillin G-Treated Embryos

Penicillin G at the concentration of 1000 μg/ml was neither embryotoxic nor teratogenic to hamster embryos. There were no significant differences between the penicillin G and the vehicle control group in any of the parameters that were tested (Table 4).

In the present study, we have confirmed the embryotoxic and teratogenic potency of VPA, $CdCl_2$, and DES in the rodent model. All three compounds produced similar effects on hamster embryos in whole embryo culture. The observed effects were growth retardation (reduced size of embryo and decreased protein content), developmental delay, and morphological abnormalities. Open neural tubes, incomplete axial rotation, irregular line of neural folds fusion, and deformed or fused somites were the most frequently observed defects.

The role of VPA in abnormal embryogenesis has been well demonstrated in rat and mouse embryo cultures [22,23,24,25]. Reported teratogenic concentration of VPA is 600–800 μM in rat embryos cultured for 48 h, whereas in mouse embryos cultured for 24 to 42 h the teratogenic concentration was 600 μM [25,26,28,29,30]. In the present study we found 60 μM to be teratogenic to hamsters in WEC. The VPA concentration used in the current study caused malformations in 25% of embryos, with a similar spectrum of abnormalities to that reported in rats and mice.

The results of this study indicate that hamster embryos were also very susceptible to cadmium chloride. This teratogen has been reported to induce defects to rat and mouse embryos *in vitro*, at concentrations ranging from 1.5 to 2.5 μM, with the highest no-effect concentration being 0.065 μM [31,32,33,34]. In the present study a concentration of 0.1 μM $CdCl_2$ induced a significant embryotoxic response (decreased protein content and head length as well as a lower morphological score) and a teratogenic effect (64% abnormal embryos). The lowest Cd concentration we tested (0.02 μM) also adversely affected the hamster embryos (decreased protein content and 7% malformed embryos).

Diethylstilbestrol is mainly known as an agent interfering with the development of the reproductive system in a fetus and is linked to uncommon vaginal cancer (clear cell adenocarcinoma) in women. Data from *in vivo* animal studies, however, indicated that this chemical is also embryotoxic. When administered orally to rats and mice during gestation, diethylstilbestrol induced prenatal death in early embryonic stage [18,19,20,33]. The present study, in which DES proved to be highly toxic to hamster embryos, is in agreement with the results of the aforementioned data. Based on our observations it seems highly probable that the severely affected embryos would die in a short time. Even at the lowest concentration, 1 μM (which was not clearly embryotoxic), neural tube defects were observed in two embryos. DES at 10 μM affected all embryonic parameters and caused malformations in 81% of hamster embryos. These findings contrast with results from rat embryo culture which showed that DES is embryotoxic and teratogenic only at concentrations above 150 μM [34].

**TABLE 4**
**Effects of Penicillin G (Negative Control) on Growth and Development of 8-day Hamster Embryos Cultured for 24 h**

| Penicillin G concentration, μg/ml | 0* | 1000 |
|---|---|---|
| No. of embryos | 16 | 14 |
| Yolk sac diameter, mm | 4.02 ± 0.21 | 3.96 ± 0.07 |
| Crown-rump length, mm | 3.14 ± 0.11 | 3.15 ± 0.07 |
| Head length, mm | 1.41 ± 0.07 | 1.36 ± 0.02 |
| Somite number | 24.8 ± 0.5 | 23.9 ± 0.4 |
| Protein content, μg/embryo | 140 ± 9 | 148 ± 11 |
| Total score | 47.9 ± 1.1 | 46.4 ± 0.5 |
| Malformed embryos, % | 0 | 0 |

* Control group from the VPA experiment.

*Note:* There were no significant differences between groups at $p < 0.05$.

Even though the whole embryo culture assay developed in 1960 has been extensively used in many developmental and toxicological laboratories for the last two decades and the vast majority of WEC experiments have been done on two animal models (rat and mouse), this assay has not been standardized yet. WEC procedures used by different laboratories still vary significantly. Conceptuses used for culture are explanted in different developmental stages, from presomite to the first somite stage. As a culture media, homologous or heterologous sera are used, frequently mixed with different synthetic culture media or supplemented with variety of micronutrients and antibiotics. Other methodological details, e.g., volume of culture medium per embryo, composition of gas mixture, and serum preparation, also differ between laboratories. All these discrepancies might have significant impact on the final results of the study and can make direct comparisons of results from different laboratories very difficult and subjective.

Embryos cultured *in vitro* are very sensitive to external conditions and investigators must optimize culture conditions in theirs laboratories when introducing WEC assay. Growth and development of embryos cultured *in vitro* should be comparable with embryos developing *in utero*.

In order to establish hamster WEC assay we initially tested different parameters of this assay including explantation procedure, developmental stage of embryos most suitable for culture, different culture media, gas equilibration, and other details. Once the culture conditions were optimized we compared development of embryos cultured *in vitro* with embryos developing *in utero;* we did not find significant differences between them.

Based on these preliminary studies we conducted experiments in which sensitivity of hamster embryos cultured *in vitro* was tested using three known teratogens. Even though the culture conditions were optimized we included a negative control, penicillin G, to make sure the obtained results are not false positive. Analysis of data from our study indicates that hamster embryos are sensitive to evaluated teratogens while they do not respond to nonteratogen, penicillin G. Comparing effective teratogenic concentrations determined in the present study with those reported in literature on rat and mouse WEC we conclude that hamster embryos cultured *in vitro* might be more susceptible to teratogenic chemicals. However it should be emphasized that comparisons of results from different laboratories in the case of WEC assay cannot be very accurate because of all possible discrepancies in applied procedures mentioned above. Based on our long experience with hamsters we find this high sensitivity of hamster conceptuses to teratogens *in vitro* similar to their susceptibility to *in vivo* teratogenesis demonstrated previously in this laboratory as well as in others [35,36,37].

One of the possible explanations for that phenomenon is that the process of embryogenesis in hamsters takes place more rapidly than in other rodent laboratory animals. For example, the hamster embryo develops from the first somite stage (Theiler's stage 12) and progresses to the limb bud stage (Theiler's stage 16) complete with a closed posterior neuropore during a 24-h period. For rat and mouse embryos to accomplish that part of development, they require culture for 48 h, a time period twice as long as in hamsters. It seems that such fast yet temporally and spatially orchestrated development can be easily disturbed by chemical stimuli.

In order to screen new chemical compounds for detection of their embryotoxic and teratogenic potentials it is necessary to use a sensitive animal model. In the present study hamster embryos cultured *in vitro* demonstrated high sensitivity to three teratogens: Cd, VPA, and DES, whereas the embryos remained unaffected by penicillin G, a nonteratogen commonly used as a negative control. Results of this study indicate that the hamster might be a good model for WEC assay. However, one needs to remember that a very susceptible model is more likely to produce false positive results. To resolve this dilemma more compounds (negative and positive teratogens) need to be tested on hamster embryos *in vitro*. Another way to distinguish the false positive from true positive teratogens (especially useful in the case of drugs) is to compare the effective embryotoxic drug concentration defined in WEC assay with the therapeutic concentration in human serum.

In closing, based on results of the present study we believe that hamster whole embryo culture is a useful additional model (beside rat and mouse) to study embryotoxic potential of chemicals. It shares all the advantages of the common laboratory rodent embryos and seems to be more susceptible to toxic stimuli. This new model will allow comparing the interspecies susceptibility to embryotoxins. Therefore, when assessing the risk to humans under certain conditions, it might be wise to consider the relative risk that a specific agent poses to different species and include experiments that take advantage of a highly susceptible animal model.

## EXPERIMENTAL METHODS

### Animals and Embryo Culture

Embryos from pregnant golden hamsters from our own outbred colony were explanted on the eighth day of gestation (when the embryos were 7.5 days old, considering the time of copulation was assumed to be around midnight). Only embryos at the 3–5 somite stage were used for culture. Explantation was performed according to previously defined methods with slight modifications [38]. The conceptuses were randomly allocated to treatment groups and transferred (four embryos per bottle) into the culture medium (4 ml) which consisted of 35% heat-inactivated hamster serum and 65% Iscove's modified or McCoy 5A medium (Gibco BRL). The bottles were continuously rotated (30 rpm) on a roller within the incubator at a temperature of 37.5°C for the 24-h culture period. The culture medium was equilibrated three times: at the start of the culture, after 8 h using a mixture of 20% $O_2$:5% $CO_2$:75% $N_2$, and after 20 h using a mixture of 40% $O_2$:5% $CO_2$:55% $N_2$.

At the end of the culture period, somite number was counted and all embryos were examined microscopically for growth, development and differentiation according to the morphological scoring system described by Brown and Fabro [39]. The yolk sac diameter (YS), crown-rump length (CR), and head length (H) were measured with the aid of a computer image analysis system. Embryo protein content was measured by the Bradford method [23].

Statistical analysis was performed using one-way ANOVA with Tukey's multiple comparisons. The level of significance was set at $p < 0.05$.

## Chemicals

2-Propylpentanoic acid (VPA) sodium salt (Sigma Chemical Co., St. Louis, MO, Cat. No. 4535) was dissolved in Tyrode's salt solution and added to the culture medium to give a final concentration of 60, 300, or 600 µM. Cadmium chloride, $CdCl_2 \cdot 2\frac{1}{2}\ H_2O$ (POCH Gliwice, min. 99.5 % purity), was dissolved in Tyrode's salt solution and added to the culture medium to give a final concentration of 0.02 µM, 0.1 µM, or 0.25 µM. In both, the VPA and cadmium experiments control embryos were grown in a medium supplemented with an equal amount of Tyrode's solution. Diethylstilbestrol (DES) (Sigma Chemical Co., St. Louis, MO, Cat. No. 4628) was dissolved in DMSO and added (2 ml/bottle) to the culture medium in order to obtain a final concentration of 1 µM, 10 µM or 120 µM. Control embryos were grown in a medium supplemented with 2 ml of DMSO per bottle. Penicillin G (Benzylpenicillin) sodium salt (Sigma Chemical Co., St. Louis, MO, Cat. No. PEN-NA) was dissolved in Tyrode's salt solution and added to the culture medium to give the final concentration of 1000 µg/ml.

## ACKNOWLEDGEMENTS

This work was supported by Grant No. 5 PO6K 022 15 from the State Committee for Scientific Research.

| | | |
|---|---|---|
| Received: | February | 1, 2001 |
| Accepted: | April | 25, 2001 |
| Published: | May | 25, 2001 |
